# Unexpected *in-situ* Free Radical Generation and Catalysis to Ag/Polymer Nanocomposite

**DOI:** 10.1038/srep11993

**Published:** 2015-07-10

**Authors:** Yifan Pang, Ruixue Wei, Jintao Wang, Liuhe Wei, Chunhui Li

**Affiliations:** 1 School of Chemistry and Molecular Engineering, Zhengzhou Key Laboratory of Elastic Sealing Materials, Zhengzhou University, Zhengzhou, 450001, China; 2 School of Materials Science and Engineering, Zhengzhou University, Zhengzhou, 450001, China

## Abstract

In this study, we discover unexpectedly that simple reaction of AgNO_3_ with oleic acid (OA) without solvent and surfactant could generate alkyl free radical which can catalyze double-bond polymerization of OA to form 1D polymeric oleic acid (POA) chain. In certain conditions, these POA chains circumvolute tightly each other to form microspheres and micro-plates in which monodisperse 4-5 nm Ag nanoparticles (NPs) were absorbed. It has been revealed that alkyl free radical generated during the redox reaction of carboxyl group of OA with Ag^+^ at relative low temperature. Then, the alkyl free radical catalyzed the double-bond polymerization of OA when the reaction temperature was further increased. Different from commonly-seen hydrophobic nanoparticles prepared in oleic acid-based microemulsion system, the nanocomposites cannot dispersed in *n*-hexane and could dispersed in ethanol and THF. The unusual dispersion behavior has been explained in terms of their structure and polarity of POA chain. The method combines the nucleation of Ag nanoparticles and the polymerization of monomer in a facile one-pot reaction, which provides a novel way for metal-polymer microsphere nanocomposite with low-cost, easy-operation and high-yield.

Nanocomposite materials consist of polymer and inorganic nanoparticles (NPs) especially noble metal NPs have shown valuable application in various fields^1^,^2^,^3^,^4^,^5^. Of them, nanocomposites containing silver NPs have received intensive research interest for its unique features in the fields of catalysis, antimicrobial agents, conducting materials and sensors^6^,^7^,^8^,^9^,^10^. In addition, silver is a good candidate in the application for surface-enhanced Raman spectroscopy and one of the best candidates for the production of metallodielectric composites in the near-infrared spectral region^11^,^12^. Therefore, considerable effort has been devoted to propose strategies to fabricate silver NPs based nanocomposites with diverse features and structures. Upon most occasions, the approaches to construct inorganic NPs-polymer nanocomposites involve multi-step and tedious separation procedures^13^,^14^. In order to confine the shell materials growth onto the particles surface region, the surface modification or anchoring of specific organic group onto the surface of NPs is required to build a bridge between the inorganic and organic matter. From the view of synthesis, the formation of inorganic NPs requires proper reducing agent and surfactants to tailor its sizes and morphologies. For the polymer domain, initiator or chain transfer agent are necessary and should be soluble in polymerization solvent^15^,^16^. Furthermore, many other strategies to attach polymer onto NPs, such as ligand exchange^17^, electrostatic interaction and soft-template *in situ* decomposition^18^,^19^, have been described. In all the above cases, the formation of NPs and the polymer growth are two independent processes and should be handled in different solution mediums. Apart from these time- and cost-consuming protocols, convenient and environmental friendly strategies for Ag/polymer nanocomposites are seldom reported. Herein, we present a novel synthetic strategy for microshperes and micro-plates of Ag/POA nanocomposites in the absence of solvent and surfactant. The formation of Ag NPs and the double-bond polymerization of oleic acid occurred in same open vessel without further post-purification.

## Results

**Figure 1:**
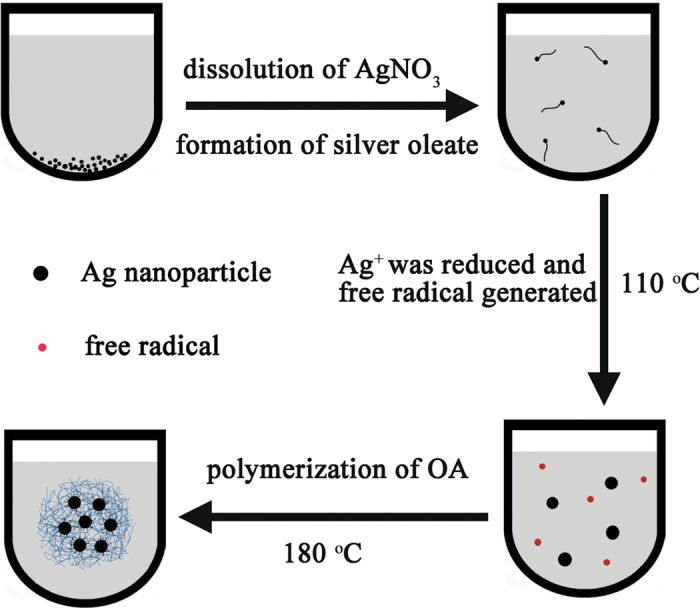
Schematic illustrating formation process of Ag/POA microspheres.

**Figure 2:**
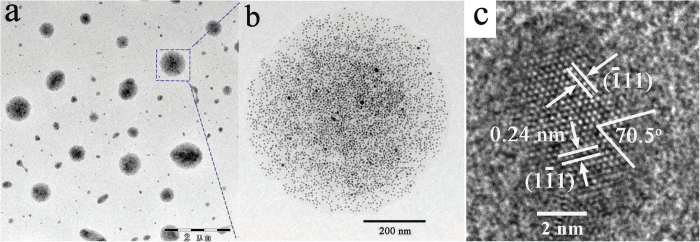
Typical TEM images of (**a**) Ag/POA microspheres and (**b**) single Ag/POA microsphere, (**c**) HRTEM image of single Ag nanoparticle.

The synthetic procedure is summarized in Fig. 1. When the system containing only AgNO_3_ powder and pure OA is heated at 110 °C for 5 hours, the colorless AgNO_3_ powder at the bottom of OA disappeared gradually and the light yellow solution turned black, indicating the formation of Ag NPs. Then the temperature was further raised to 180 °C and stirred for 4 hours. After the reaction, *n*-hexane was added to precipitate the product. Notably, the as-obtained product show unusual dispersibility. Contrary to other commonly-seen nanomaterials prepared in OA-based solution, the as-obtained nanocomposites can be dispersed in ethanol, but not in nonpolar solvents, such as *n*-hexane and cyclohexane. The “blue lines” in Fig. 1 means the POA chains and the microsphere is a diagrammatic sketch of TEM image shown in Fig. 2b. It is worthy to note that the POA chains in reaction OA system could form spherical structure with the addition of hexane for it is insoluble in hexane. Single POA chain is too slim to observe directly with TEM technique but could be seen when it is entwined to form bigger microspheres or nanowires.

As shown in Fig. 2a,b, monodisperse 4-5 nm Ag NPs exist in the region of low-contrast microspheres with variable sizes. Powder X-ray diffraction (XRD) measurements were used to identify the internal crystalline structures of as-prepared products, as shown in Fig. S1. Figure 2c shows a representative HRTEM image of a single NP of fcc Ag, the lattice spacing along different direction of 0.24 nm correspond well to the crystal plane of (111) and the angle between two lattice direction is 70.5°. Thus, the exposed face of the single NP is (220) plane. The formation of Ag/POA consists of two reaction processes conducted at 110 and 180 °C, respectively. At relatively low temperature, Ag^+^ ions were reduced by the carboxyl groups of OA to yield uniform 4-5 nm Ag NPs, as shown in Fig. S2. If the reaction was stopped and adding NaCl solution to the solution, there would not give any white precipitate, indicating that all Ag^+^ ions have been reduced to Ag particles. Like most NPs prepared in OA-based system, the Ag NPs can be deposited in ethanol and dispersed in *n*-hexane, which has been widely comprehended for the oleate ligand absorbed onto the surface of Ag NPs. In this work, we further increased the temperature to 180 °C and obtained Ag/POA nanocomposite with unusual dispersibility. The interesting difference of dispersibility has not been reported and cannot be explained in the light of molecular structure of oleic acid as expounded in the past.

## Discussion

Two controlled experiments have been performed to clarify the formation mechanism of Ag/POA. Firstly, AgNO_3_ cannot react with octadecane even heated at 180 °C for hours, indicating that Ag NPs merely came from the reduction of Ag^+^ ions by carboxyl groups of OA and was irrelevant to the 18-C fatty chain. Second, if AgNO_3_ was mixed with octadecanoic acid instead of OA and heated at 180 °C for hours, the dispersibility of product is not dispersive in ethanol but is dispersive in *n*-hexane. TEM results revealed that the product was single NPs, but no microspheres as shown in Fig. 2a. Considering that the only difference between octadecanoic acid and OA is the double-bond, it can be alleged that the double-bond is a crucial factor to the Ag/POA hybrid microspheres formation and their unusual dispersibility.

**Figure 3:**
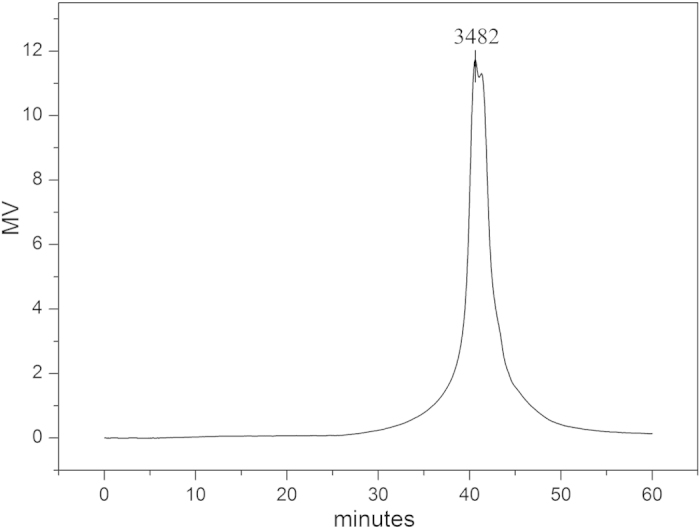
GPC chromatogram of POA.

Based on the TEM image of microsphere (Fig. 2a) and above experimental facts, we concluded that the microspheres were indeed 1D polymeric oleic acid chains formed through the double-bond polymerization. The polymer has been characterized by gel permeation chromatography (GPC), which supported our deduction (Fig. 3 and Table 1). The *M*_n_ value of the POA dissociated from the Ag/POA hybrids was 4422, and the polydispersity index *M*_W_/*M*_n_ was 1.863, according to GPC. TEM images shown that the Ag nanoparticles were wrapped in the microspheres composed of polymer chains (Fig. 2a). As is known to all, it is difficult for oleic acid to self-polymerization due to the double-bond located in the middle of the oleic acid molecule. So, what is the cause of the polymerization? Several experiments have been conducted to clarify the cause of the polymerization.

**Figure 4:**
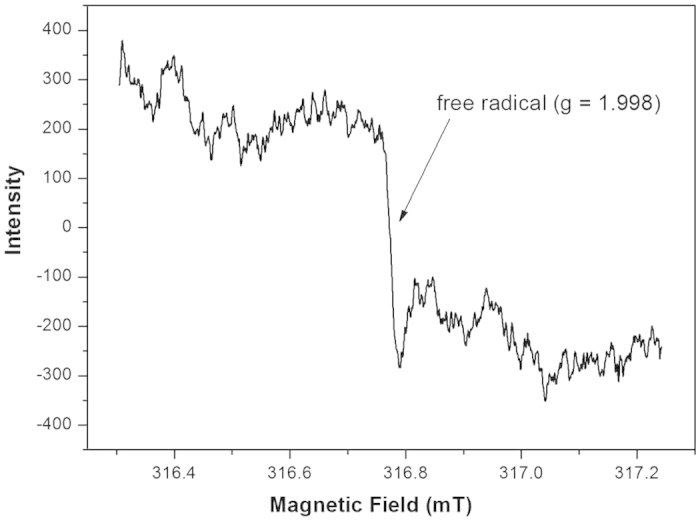
ESR spectra of OA solution after reduction of Ag^+^.

**Figure 5:**
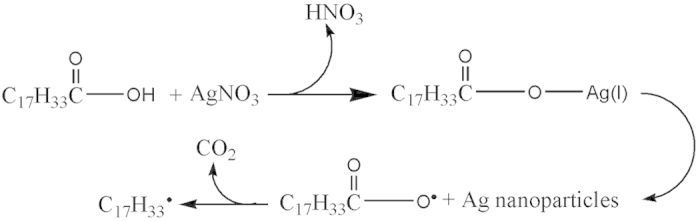
Proposed formation mechanism of free radical.

The precipitated Ag NPs obtained at 110 °C stage could be easily dispersed in OA for its low polarity. After heating at 180 °C for 4 h in fresh OA, the product still can be deposited in ethanol and dispersed in nonpolar solvents, which is totally different from the Ag/POA microspheres. The TEM image and GPC measurement shown that polymerization did not happen. Based on these, it is reasonable to conclude that there is “organic free radical” existing in the original OA solution which was produced at the redox reaction stage and initiated the double-bond polymerization of OA. ESR spectrum is often adopted to detect the existence of free radical. In our experiments, Ag nanoparticles with delocalized electron cannot give ESR signal and only single electron from molecular orbital is possible to produce ESR signal. The original OA solution conducted at room temperature exhibited an ESR signal at approximately 323 mT (g = 1.998), as shown in Fig. 4, which verified our conjecture. While the detailed mechanism is not clear at this moment, a tentative mechanism is proposed as shown in Fig. 5. The reaction between AgNO_3_ and OA leads to the generation of silver oleate followed by the reduction of Ag^+^ and the formation of carboxyl radical, which undergoes fast decarboxylation to generate the alkyl radical^20^.

**Figure 6:**
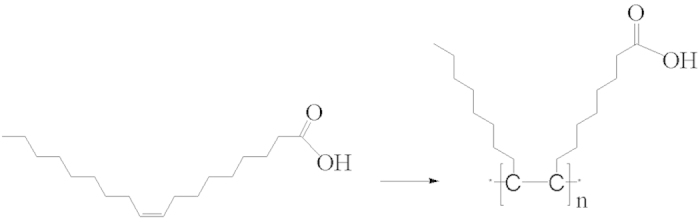
Polymerization of OA initiated by alkyl free radicals.

The double-bond polymerization of OA was initiated by the alkyl radicals to form chain-like POA polymer (Fig. 6). IR spectra of Ag/POA is given in Fig. S3. Two strong peaks around 2828 and 2855 cm^−1^ can be attributed to the asymmetry and symmetry stretching vibration of C–H bond. Two strong peaks at 1564 and 1390 cm^−1^ were caused by the asymmetry and symmetry stretching vibration of −COOH group. All these peaks give direct support to the existence of POA^21^ The dispersibility of Ag/POA can also be explained in term of the structure of POA. Owing to the polarity of carboxyl group, the as-prepared Ag/POA is dispersive in polar solvent such as THF and ethanol, and precipitated in nonpolar solvent such as n-hexane.

**Figure 7:**
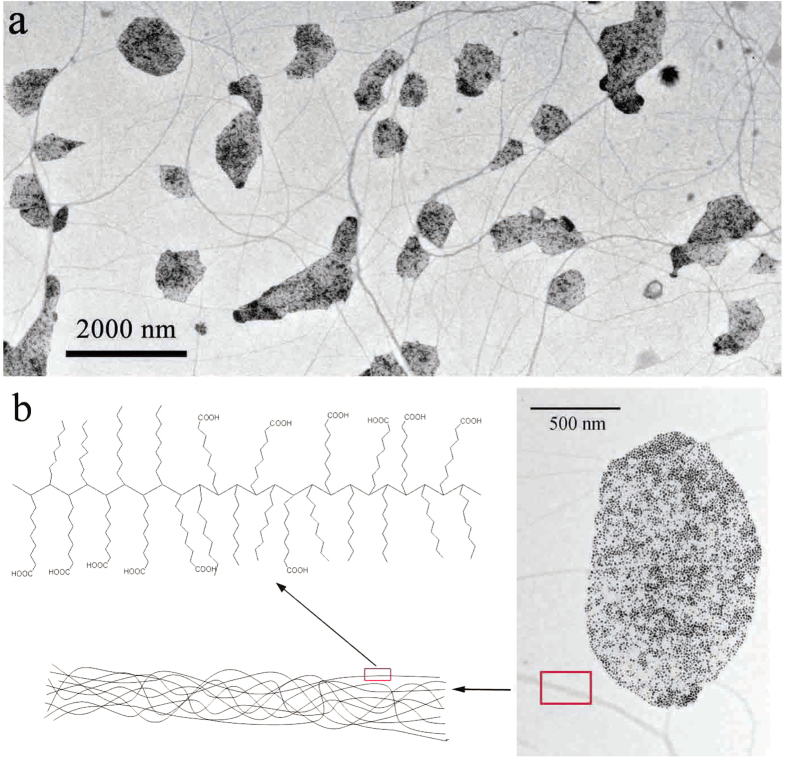
(**a**) POA nanowires in TEM images of swollen Ag/POA nanomaterials in EtOH; (**b**) Schematic representation of POA chain structure.

Based on above analysis, it can be concluded that Ag/POA microspheres were entangled polymerized OA chains and monodisperse Ag NPs were involved inside. Because of plenty of carboxyl groups and octyl groups, Ag/POA can be dispersed in solvents such as ethanol and THF and precipitated in non-polar solvents such as hexane and strong-polar water. The entwined POA chains formed spherical POA structure because of it is insoluble in hexane and there is no strong covalent bond among those POA chains. So, compared with other polymer spheres synthesized using crosslinker, the shapes of Ag/POA are more flexible and can be swollen in good solvents such as THF and ethanol to release free POA chains and single Ag nanoparticles. In our experiments, ethanol was chosen to swell Ag/POA structure for Ag nanoparticles coated with oleate could not dispersed in it and could be removed by centrifugation while slightly swollen Ag/POA and POA chains were still existed in ethanol solution. TEM images show that all spherical Ag/POA deformed to plate-like hybrid, indicating that the structure of circumvolute tightly POA microspheres became loose because of the soaking of solvent (Fig. 7). Additional, numerous POA chains separated from POA spheres and intertwined each other to form POA nanowires because of the hydrogen bond interaction of carboxyl groups of such polymer chains. The flexible low-contrast nanowires shown in Fig. 7 were another evidence to support the polymerization of OA. It should be noted that the synthetic strategy is general and can be extended to design novel hybrid nanocomposites via the direct reaction between the AgNO_3_ powder and other organic acid containing double-bond such as 11-undecylenic acid, shown in Fig. S4.

In conclusion, the Ag/POA core-shell nanocomposites were synthesized by direct reaction between OA and AgNO_3_. Detailed research showed that the microspheres were composed of entangled POA chains containing monodisperse Ag nanoparticles. It should be noted that the generation of alkyl free radical during the redox process and its catalytic activity for double-bond polymerization have been ignored up to now. Interestingly, only when heated at higher temperature (180 °C for OA), the free radicals could initiate the double-bond polymerization to form one-dimensional POA chains. Taking into account the steric hindrance of OA, the catalytic activity of the alkyl free radicals are comparably high and the method can be expanded to initiate those monomers that are difficult to polymerize. The generation of the free radical during the redox reaction between metal salts and carboxylic acids and their catalytic activity can help us comprehend deeper the reaction mechanism, which is suggestive to design novel metal/polymer core-shell nanostructures.

## Methods

### General experimental detail

The reagents used in this work, including ethanol and THF were of analytical grade from the Beijing Chemical Factory of China. All the reagents were used without further purification. The crystal structure and phase purity were characterized by using a X’Pert PRO X-ray diffractometer (XRD) with Cu Kα radiation (λ = 1.5406 Å) in the 2*θ* range 5° to 90°. The Fourier transform infrared (FT-IR) spectra of the sample were collected on a PerkinElmer Spectrum GX from 4000 to 400 cm^−1^. High-resolution transmission electron microscopy (HRTEM) was performed using a FEI Tecnai G2 F20 S-Twin microscope working at an acceleration voltage of 200 kV. TEM was performed using a JEM-1200EX microscope working at an acceleration voltage of 120 kV. ESR spectrum was conducted using FA-200 ESR spectrometer. The molecular weights of the products were measured by GPC on a HLC-8320GPC (TOSOH, EcoSEC GPC System) system at 40 °C with THF as mobile phase at a flow rate of 0.6 ml/min.

### Synthesis of Ag/POA hybrid nanocomposite

In a typical synthesis of Ag/POA nanomaterials, 0.5 mmol AgNO_3_ powder was added into 10 mL oleic acid (OA). The reaction was heated at 110 °C with vigorous stirring. After 5 h, the black mixture was heated at 180 °C. Four hours later, the products were collected by adding 20 mL *n*-hexane and washed with ethanol and *n*-hexane several times. The as-obtained Ag/POA nanomaterials were dispersed in 5 mL of ethanol for TEM measurement.

### Preparation of POA for GPC measurement

The as-obtained Ag/POA (10 mg) was mixed with 40 mL dilute HNO_3_ (0.05M) and then transferred into a 50-mL autoclave, sealed, and heated at 60 °C for about 30 h. The system was then allowed to cool to room temperature and the solution was dried at 70 °C. 20 mL THF added to dissolve the polymer followed by filtration. The filtrate was collected and vacuumed to dry.

### Preparation of swollen Ag/POA for TEM observation

A copper grid covered with an amorphous carbon film was placed on a filter paper. The as-obtained Ag/POA (10 mg) and 20 mL ethanol were mixed together. After stirring overnight, the system was centrifuged to remove dissociative Ag nanoparticles and a solution droplet was then transferred to the film surface.

## Additional Information

**How to cite this article**: Pang, Y. *et al*. Unexpected *in-situ* Free Radical Generation and Catalysis to Ag/polymer Nanocomposite. *Sci. Rep*. **5**, 11993; doi: 10.1038/srep11993 (2015).

## Supplementary Material

Supplementary Information

## Figures and Tables

**Table 1:** GPC Data for POA.

R. T. (min.)	*M*_n_	*M*_w_	*M*_p_	*M*_z_	PDI
40.617	4422	8239	3482	27038	1.863

## References

[b1] Liu H. Z. . Laundering durable antibacterial cotton fabrics grafted with pomegranate-shaped polymer wrapped in silver nanoparticle aggregations. Sci. Rep. 4, 5920–5928 (2014). 25082297 10.1038/srep05920 PMC4118188

[b2] Ge J. P. & Yin Y. D. Responsive Photonic Crystals. Angew. Chem. Int. Ed. 50, 1492–1522 (2011). 10.1002/anie.200907091 21254316

[b3] Xu S. . Toward Designer Magnetite/Polystyrene Colloidal Composite Microspheres with Controllable Nanostructures and Desirable Surface Functionalities. Langmuir 28, 3271–3278 (2012). 22288525 10.1021/la2043137

[b4] Liu Y., Yin J. J. & Nie Z. H. Harnessing the Collective Properties of Nanoparticle Ensembles for Cancer Theranostics. Nano Res. 7, 1719–1730 (2014).

[b5] Ku K. H. . Size-Controlled Nanoparticle-Guided Assembly of Block Copolymers for Convex Lens-Shaped Partcles. J. Am. Chem. Soc. 136, 9982–9989 (2014). 24927484 10.1021/ja502075f

[b6] Luo S. B., Yu S. H., Sun R. & Wong C. P. Nano Ag-Deposited BaTiO_3_ Hybrid Particles as Fillers for Polymeric Dielectric Composites: Toward High Dielectric Constant and Suppressed Loss. ACS Appl. Mater. Interfaces 6, 176–182 (2014). 24320940 10.1021/am404556c

[b7] Lu H. F., Zhang D., Ren X. G., Liu J. & Choy W. C. H. Selective Growth and Integration of Silver Nanoparticles on Silver Nanowires at Room Conditions for Transparent Nano-Network Electrode. ACS Nano 8, 10980–10987 (2014). 25285984 10.1021/nn504969z

[b8] Li W., Sun J. & Chen M. F. Triboelectric Nanogenerator using Nano-Ag Ink as Electrode Material. Nano Energy 3, 95–101 (2014).

[b9] Ye S. J. & Lu Y. Optical Properties of Ag@Polypyrrole Nanoparticles Calculated by Mie Theory. J. Phys. Chem. C 112, 8767–8772 (2008).

[b10] Lu W. B., Cao X. W., Tao L., Ge J. & Qian W. P. A Novel Lable-Free Amperometric Immunosensor for Carcinoembryonic Antigen Based on Ag Nanoparticle Derorated Infinite Coordination Polymer Fibres. Biosens. Bioelectron. 57, 219–225 (2014). 24583695 10.1016/j.bios.2014.02.027

[b11] Ye S. J., Chen S. J., Wang H. J. & Lu Y. pH-Responsive Luminescent Properties of Ag@PPy Nanoparticles. Phys. Chem. Chem. Phys. 13, 4668–4673 (2011). 21279225 10.1039/c0cp02184a

[b12] Liu T. M. . One-step shell polymerization of inorganic nanoparticles and their applications in SERS/nonlinear optical imaging, drug delivery, and catalysis. Sci. Rep. 4, 5543–5552 (2014). 24998932 10.1038/srep05593 PMC4083277

[b13] Wu T., Zhang Y. F., Wang X. F. & Liu S. Y. Fabrication of Hybrid Silica Nanoparticles Densely Grafted with Thermoresponsive Poly(N-isopropylacylamide) Brushes of Controlled Thickness via Surface-Initiated Atom Transfer Radical Polymerization. Chem. Mater. 20, 101–109 (2008).

[b14] Li C. Z. & Benicewicz B. C. Synthesis of Well-defined Polymer Brushed Grafted onto Silica Nanoparticles via Surface Reversible Addition-Fragmentation Chain Transfer Polymerization. Macromolecules 38, 5929–5936 (2005).

[b15] Matsuno R., Yamamoto K., Otsuka H. & Takahara A. Polystyrene- and Poly(3-vinylpyridine)-Grafted Magnetite Nanoparticles Prepared through Surface-Initiated Nitroxide-Mediated Radical Polymerization. Macromolecules 37, 2203–2209 (2004).

[b16] Pei X. W., Hao J. C. & Liu W. M. Preparation and Characterization of Carbon Nanotubes-Polymer/Ag Hybrid Nanocomposites via Surface RAFT Polymerization. J. Phys. Chem. C 111, 2947–2952 (2007).

[b17] Narain R., Gonzales M., Hoffman A. S., Stayton P. S. & Krishnan K. M. Synthesis of Monodisperse Biotinylated P(NIPAAm)-Coated Iron Oxide Magnetic Nanoparticles and their Bioconjugation to Streptavidin. Langmuir 23, 6299–6304 (2007). 17451262 10.1021/la700268g

[b18] Shen Y. . Gold Nanoparticles Coated with a Thermosensitive Hyperbranched Polyelectrolyte: Towards Smart Temperature and pH Nanosensors. Angew. Chem. Int. Ed. 47, 2227–2230 (2008). 10.1002/anie.200704572 18275053

[b19] Lai J. J. . Dual Magnetic-/temperature-Responsive Nanoparticles for Microfluidic Separation and Assays. Langmuir 23, 7385–7391 (2007). 17503854 10.1021/la062527g

[b20] Wang Z. T. . Silver-Catalyzed Decarboxylative Chlorination of Aliphatic Carboxylic Acids. J. Am. Chem. Soc. 134, 4258–4263 (2012). 22316183 10.1021/ja210361z

[b21] Li C. H., Peng Q. & Li Y. D. Controlled Synthesis of Nearly Monodispersed Mn_2_(PO_4_)Cl Nanocrystals and Nanorods. Cryst. Growth Des. 8, 243–246 (2008).

