# Supplementary material for: Unexpected in-situ Free Radical Generation and Catalysis to Ag/Polymer Nanocomposite
Source: Sci Rep. 2015 Jul 10;5:11993. doi: 10.1038/srep11993 (PMC4498183; doi:10.1038/srep11993)
Supplement: Supplementary Information [file srep11993-s1.pdf]

Supporting Information

Unexpected in-situ Free Radical Generation and Catalysis to Ag/polymer Nanocomposite

Yifan Pang,[1] Ruixue Wei,[1] Jintao Wang,[2] Liuhe Wei,[1] Chunhui Li,[1,*]

[1]School of Chemistry and Molecular Engineering, Zhengzhou Key Laboratory of Elastic Sealing Materials, Zhengzhou University, Zhengzhou, 450001, China, [2]School of Materials Science and Engineering, Zhengzhou University, Zhengzhou, 450001, China.

Content

1. XRD patterns of as-obtained Ag/POA nanocomposites
2. TEM image of Ag nanoparticles prior to 180°C treatment
3. IR spectra of of Ag/POA
4. TEM images of Ag/polymer using 11-undecylenic acid instead of OA

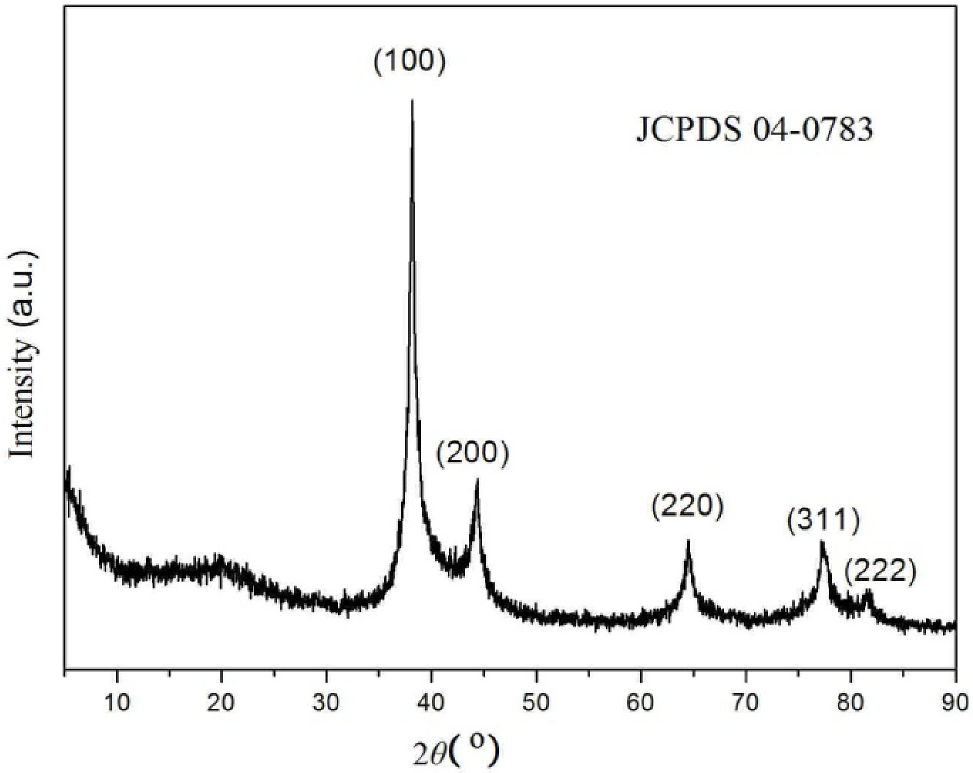

Figure S1. XRD patterns of as-obtained Ag/POA nanocomposites

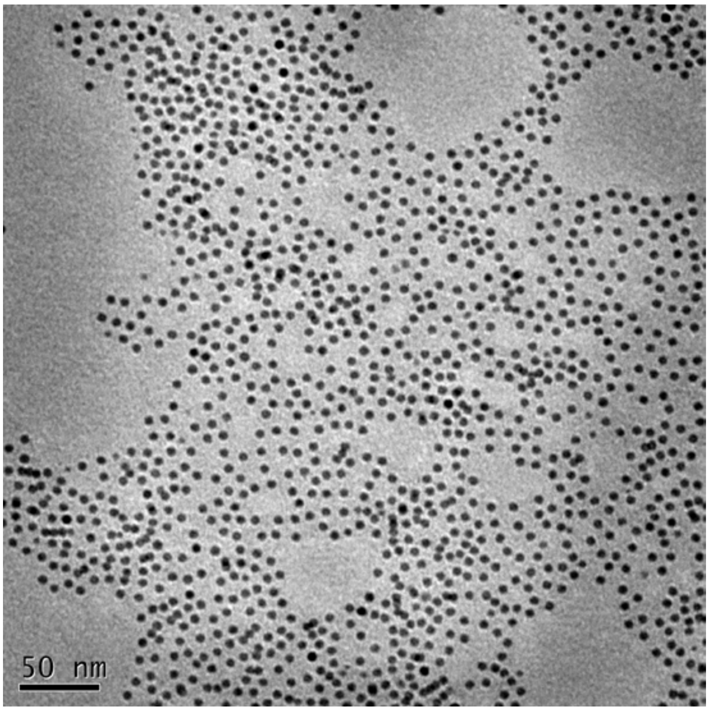

Figure S2. TEM image of Ag nanoparticles prior to 180°C treatment.

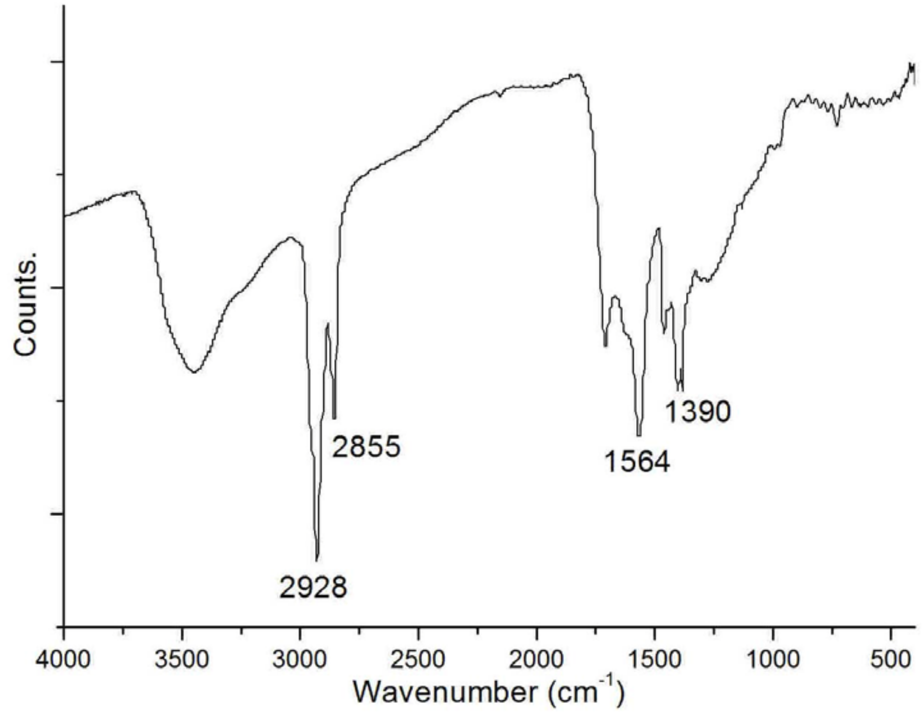

Figure S3. IR spectra of of Ag/POA.

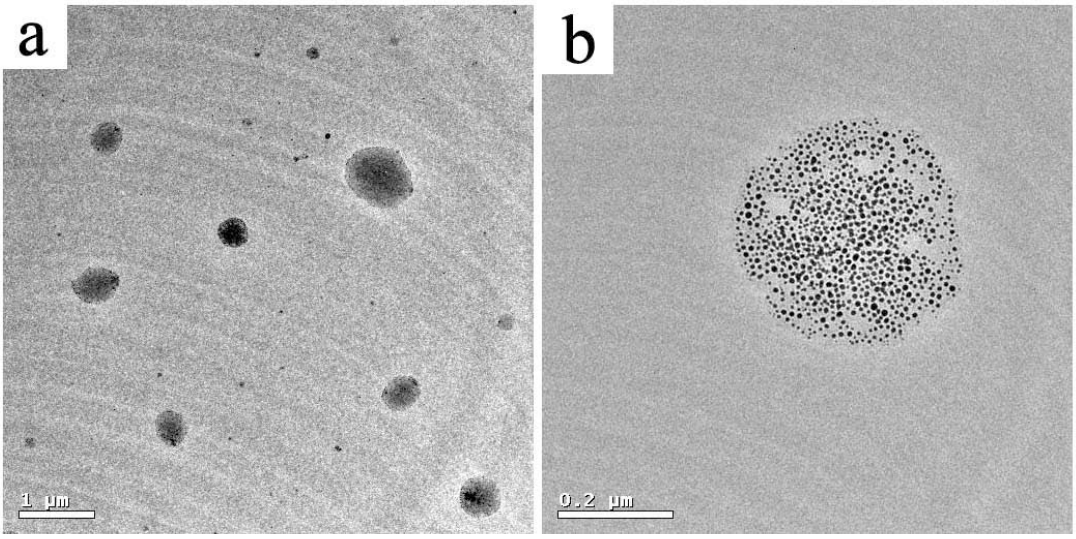

Figure S4. TEM images of Ag/polymer using 11-undecylenic acid instead of OA.
